# Secondary zoonotic dog-to-human transmission of SARS-CoV-2 suggested by timeline but refuted by viral genome sequencing

**DOI:** 10.1007/s15010-022-01902-y

**Published:** 2022-08-20

**Authors:** John M. Hoppe, Louise U. Füeßl, Katrin Hartmann, Regina Hofmann-Lehmann, Alexander Graf, Stefan Krebs, Helmut Blum, Irina Badell, Oliver T. Keppler, Maximilian Muenchhoff

**Affiliations:** 1 Medizinische Klinik und Poliklinik IV, Division of Nephrology, LMU Klinikum, Munich, Germany; 2 Medizinische Kleintierklinik, Zentrum für Klinische Tiermedizin, LMU München, Munich, Germany; 3 Clinical Laboratory, Department of Clinical Diagnostics and Services, and Center for Clinical Studies, Vetsuisse Faculty, University of Zurich, Zurich, Switzerland; 4 Laboratory for Functional Genome Analysis, Gene Center, LMU München, Munich, Germany; 5 Max von Pettenkofer Institute and Gene Center, Virology, National Reference Center for Retroviruses, LMU München, Munich, Germany; 6 German Center for Infection Research (DZIF), Partner Site, Munich, Germany

**Keywords:** SARS-CoV-2, Reverse zoonosis, Zoonotic spillover, Secondary zoonosis, COVID-19, Canine, Sequencing, RT-PCR, Antibodies

## Abstract

**Purpose:**

The risk of secondary zoonotic transmission of SARS-CoV-2 from pet animals remains unclear. Here, we report on a 44 year old Caucasian male presenting to our clinic with COVID-19 pneumonia, who reported that his dog displayed respiratory signs shortly prior to his infection. The dog tested real-time-PCR (RT-PCR) positive for SARS-CoV-2 RNA and the timeline of events suggested a transmission from the dog to the patient.

**Methods:**

RT-PCR and serological assays were used to confirm SARS-CoV-2 infection in the nasopharyngeal tract in the dog and the patient. We performed SARS-CoV-2-targeted amplicon-based next generation sequencing of respiratory samples from the dog and patient for sequence comparisons.

**Results:**

SARS-CoV-2 infection of the dog was confirmed by three independent PCR-positive pharyngeal swabs and subsequent seroconversion. Sequence analysis identified two separate SARS-CoV-2 lineages in the canine and the patient’s respiratory samples. The timeline strongly suggested dog-to-human transmission, yet due to the genetic distance of the canine and the patient’s samples paired-transmission was highly unlikely.

**Conclusion:**

The results of this case support current knowledge about the low risk of secondary zoonotic dog-to-human transmissions of SARS-CoV-2 and emphasizes the strength of genomic sequencing in deciphering viral transmission chains.

**Supplementary Information:**

The online version contains supplementary material available at 10.1007/s15010-022-01902-y.

## Introduction

Severe acute respiratory syndrome coronavirus type 2 (SARS-CoV-2) is a Betacoronavirus that was first reported in late 2019 to cause clusters of severe respiratory illness in Wuhan, China [1]. By January 2020, the virus had been isolated and sequenced [2, 3] and after spreading to multiple countries across the globe causing coronavirus disease 2019 (COVID-19) leading to a significant number of fatalities, the World Health Organization (WHO) announced on March 11, 2020 that COVID-19 should be characterized as a global pandemic [4]. Besides human-to-human transmission, natural reverse zoonotic transmissions from humans to various animal species have been described [5]. These include a few cases of natural human-to-dog transmissions [6, 7]. However, screening of more than 6000 canine, feline and equine specimens in the United States, Europe, Canada and South Korea from areas experiencing a significant number of human COVID-19 cases, all animal specimens tested negative [8]. In another study analyzing a total of 2257 oropharyngeal and nasal swab specimen from 877 dogs and 260 cats (including 18 animals from COVID-19-affected households and 92 animals with signs of respiratory disease) from Southern Germany and Northern Italy during the first wave of the COVID-19 pandemic (March to July 2020) for the presence of SARS-CoV-2 RNA using RT-PCR, none of the dogs was confirmed positive. Analyses of convenience sera from 118 animals identified one dog from Lombardy, as positive for anti-SARS-CoV-2 receptor binding domain (RBD) antibodies and neutralizing activity [9]. These findings support the hypothesis that the prevalence of SARS-CoV-2 infection in pet dog populations is low and human-to-animal transmission is very rare. Despite identification of a few natural cases of SARS-CoV-2 infection in dogs, there are no descriptions of secondary zoonotic transmissions from dogs to humans. To our knowledge, there have only been two reports of assumed secondary zoonotic transmissions, mink-to-human on a mink farm and hamster-to-human in a pet store [10, 11]. Here, we describe a SARS-CoV-2-infection in a COVID-19 patient and his dog. In this case, dog-to-human transmission of SARS-CoV-2 was suggested by the chronological order of events. However, phylogenetic analyses revealed that the SARS-CoV-2 sequences of the dog and the patient were genetically distinct and transmission likely occurred from two separate sources.

## Materials and methods

### Sample collection

**Fig. 1:**
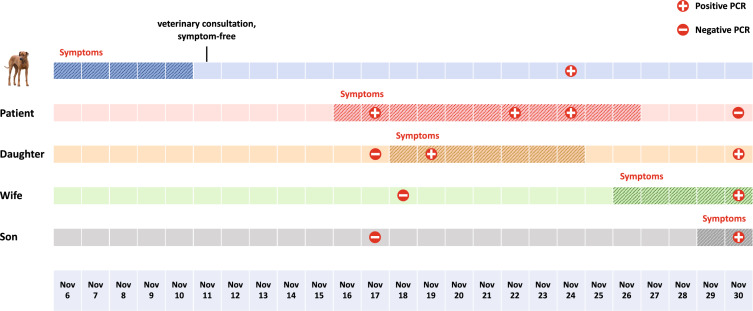
Chronological order of onset of symptoms and SARS-CoV-2 PCR test results in the dog and family members. Infection cluster timeline of COVID-19 symptoms and reverse-transcriptase–polymerase-chain-reaction (RT-PCR) detection of SARS-CoV-2 RNA in nasopharyngeal swabs of the patient, family members and family dog

Nasopharyngeal swabs from the patient and dog were obtained at various time points (Fig. 1). All nasal swabs were immersed in viral transport medium (Copan diagnostics, Murrieta, USA) and transported to the Department of Virology at the Pettenkofer-Institute, LMU Munich. Nasopharyngeal swab collection of the other family members and analysis were performed at a non-LMU SARS-CoV-2 test center in Munich. Blood samples of the dog were obtained in November 2020 and March 2021. Serum samples were stored at − 20 °C and transported on dry ice to the Clinical Laboratory, Department of Clinical Diagnostics and Services, and Center for Clinical Studies of the University of Zurich, Switzerland. Human blood samples were analyzed at the institute for laboratory medicine, LMU Klinikum. For sequence comparison of the dog and the patient to characterize the different SARS-CoV-2 strains circulating in Munich at that time, routine respiratory samples sent to the diagnostic laboratory at the Pettenkofer-Institute between September and December 2020 that tested positive by PCR for SARS-CoV-2-RNA (*n* = 549) were used.

### Antibody testing

Serum samples of the dog were tested for SARS-CoV-2 specific antibodies via receptor binding domain enzyme-linked immunosorbent assay (RBD-ELISA) and surrogate viral neutralization test (sVNT) as described previously [9]. Human serum samples were tested for anti-nucleocapsid antibodies by standard commercial assays (Euroimmune, Lübeck, Germany).

### Molecular detection and quantification of SARS-CoV-2

Nasopharyngeal swab samples were tested by reverse transcription polymerase-chain-reaction (RT-PCR) using the cobas^®^ 6800 SARS-CoV-2 Test (Roche Diagnostics, Mannheim, Germany). Samples with a cycle threshold value (Ct) of less than 40 were regarded positive. Results of the nucleocapsid (N) reaction were used for quantification using standard curves based on serial dilutions in duplicate of a quantitative reference standard (INSTAND e.V.) as described previously [12]. Viral loads were expressed as SARS-CoV-2 N-gene copy numbers per ml of transport medium.

### SARS-CoV-2 genome sequencing

Viral ribonucleic acid (RNA) was extracted from nasopharyngeal swab samples using the Qiasymphony DSP Virus/Pathogen kit on a Qiasymphony extraction robot (Qiagen, Hilden, Germany). The extracted RNA was translated into cDNA using the SuperScript III First-Strand Synthesis System (Invitrogen, Thermo Fisher Scientific, Dreieich, Germany). Amplicon pools covering the SARS-CoV-2 genome were prepared using the ARTIC network nCoV-2019 sequencing protocol v2 [13]. Briefly, amplicon pools were diluted to 0.2 ng/µl and tagmented with nextera XT library prep kit (Illumina, San Diego, USA). Nextera libraries were dual-barcoded and sequenced on an Illumina Hiseq 1500 instrument. Sequence data were analyzed based on the Artic bioinformatics pipeline as reported previously [14]. The consensus sequences and associated sample metadata were uploaded to the GISAID repository (see accession numbers in Supplementary Table 1).

### Phylogenetic analysis and heatmap illustration

The phylogenetic tree was obtained from a local Auspice installation using the Snakemake workflow [15]. Briefly, the workflow filters genomes based on pre-defined criteria, such as quality and lengths, aligns the genomes to the reference genome and constructs the phylogenetic tree, based on a maximum-likelihood approach. The mutation frequency heatmaps were generated with the variant frequencies obtained from Freebayes using the R package pheatmap [16]. Reported variants had to show a minimal coverage of 20 reads and a variant frequency of at least 40% in one of the samples.

### Ethical approval

The patient gave informed consent to publication of all medical and personal data regarding this case. Further, informed consent was given for dog sample collection and collection of data on SARS-CoV-2 test results for all family members. Data were pseudonymized for the analysis. Analysis of the sequence data obtained from the samples sent to the Pettenkofer-Institute between September and December 2020 was approved by the local ethics committee (reference number 21-0740).

### Case history

A 44-year old previously healthy Caucasian male presented to our emergency department with dyspnea, elevated respiratory rate (24/min), fever and cough. Infection with SARS-CoV-2 was confirmed by RT-PCR. In addition, later testing showed reactivity for SARS-CoV-2 antibodies. Initial blood analysis revealed elevated inflammatory markers, such as C-reactive protein (CRP) of 4.1 mg/dL (normal range < 0.5 mg/dL) and leukocytosis 11.7 G/L (normal range 3.9 to 9.8 G/L) with left shift. Without oxygen demand the patient was transferred to the COVID-19 ward. Throughout the hospital stay, multiple nasopharyngeal swabs of the patient, collected on day 2, 7 and 9 after onset of symptoms, were positive for SARS-CoV-2 on RT-PCR. Furthermore, seroconversion for SARS-CoV-2 specific antibodies was detected (Euroimmune assay, Lübeck, Germany). After 11 days of hospitalization during which the patient had displayed severe cough and mild fever, the patient’s condition had improved substantially, allowing for hospital discharge.

The patient credibly denied any contact to a person with confirmed COVID-19 prior to his infection. However, he noted that his dog, a nine month-old female Rhodesian Ridgeback, had started displaying cough and fatigue 11 days prior to the patient’s illness (see timeline, Fig. 1). During the peak of the second pandemic wave in Munich, the family kept the dog indoors, except for short walks. Hence, the dog was in close contact with all family members. The dog developed clinical signs on November 6th 2020, which then persisted for five days. A day after respiratory signs had subsided, the dog was presented to a veterinary who suspected that the dog suffered from canine infectious respiratory disease (CIRD) complex, although it had been vaccinated against the same pathogens involved in CIRD a few months prior to the onset of clinical signs. On November 16th, 6 days after the dog had stopped to show clinical signs, the patient developed symptoms and tested positive for SARS-CoV-2 on November 17th.

**Fig. 2:**
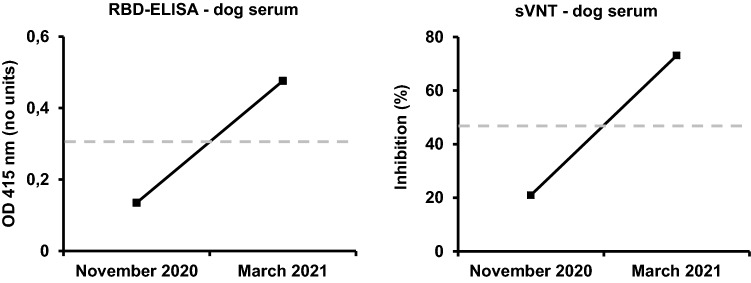
Antibody detection in canine serum samples. Analysis of canine serum samples for detection of SARS-CoV-2-specific antibodies collected in November 2020 and March 2021. **a** Antibodies against the receptor binding domain (RBD) measured by enzyme-linked immunosorbent assay (ELISA) in values of optical density measured at 415 nm (OD 415 nm). **b** Percentage of virus inhibition measured by the surrogate virus neutralization test (sVNT). Dashed lines (gray) indicate in (**a**) the threshold for test positivity at optical density of 0.3 and in (**b**) the positive cut-off value at 47% inhibition

For further investigation on a possible secondary zoonotic event, three independent nasopharyngeal swabs were obtained from the dog on November 24th, eight days after the patient’s admission to our hospital and two weeks after the dog last displayed clinical signs. All three swab samples tested positive for SARS-CoV-2 RNA by RT-PCR (Ct-values 35.5, 33.5 and 33.9, respectively) (Supplementary table 1). To further confirm SARS-CoV-2 infection of the dog, serum samples were collected in November 2020 and at a follow-up visit in March 2021. Both, RBD-ELISA and sVNT showed seroconversion for SARS-CoV-2 specific antibodies at the follow-up visit, confirming canine SARS-CoV-2 infection (Fig. 2).

For further analysis of other possible transmission pathways, data on symptoms and SARS-CoV-2 testing were collected for all other members of the infection cluster (wife, daughter and son). At the time when the patient first tested positive, all other family members tested negative. Then, on November 18th 2020, the daughter developed mild respiratory symptoms and tested positive shortly thereafter. On November 25 and 29th, the wife and son also developed symptoms, respectively, and both tested positive on November 30th. Markedly, the chronology of symptoms within the infection cluster suggested dog-to-human transmission of SARS-CoV-2 as the dog was first to display clinical signs.

## Results

To test the hypothesis that transmission occurred from the dog to the patient, we used next generation sequencing (NGS) to generate near-full length SARS-CoV-2 genomes from the three canine swab samples and two samples of the patient. The other family members were tested at a different laboratory and therefore not sequenced. Genome coverage of the two patient’s samples was very good (99.9%), while coverage for the three dog samples was lower (78–85%) as expected given the higher Ct-values of these samples (Supplementary Table 1).

**Fig. 3:**
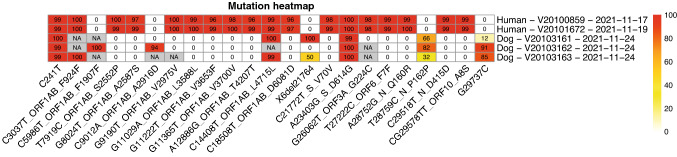
SARS-CoV-2 sequence comparison between canine and human samples. The heatmap shows the frequencies of mutations in SARS-CoV-2 sequences obtained from canine and human swab samples in comparison to the reference genome (Wuhan-Hu-1). Genome positions with insufficient sequencing coverage (less than 20 reads at that position) are indicated as “NA”. The label for each mutation on the x-axis first indicates the mutation on the nucleotide level, then the location within a protein coding gene if applicable and its effect on the amino acid level. For example, A28752G_N_Q160R indicates a substitution of adenosine to guanosine at genome position 28,752 corresponding to an exchange from glutamine to arginine at position 160 in the nucleocapsid protein. Sample identifiers and collection dates are indicated on the *y*-axis

Nevertheless, genome coverage was sufficient to perform sequence comparisons between the dog and the patient’s samples (Fig. 3). Unexpectedly, the sequences of the dog and the patient were divergent by multiple single nucleotide polymorphisms (SNPs). In canine samples, we detected SNPs compared to the reference genome (Wuhan-Hu-1) at five genome positions that were not present in the patient’s samples. Conversely, we detected 14 SNPs that were present in the patient’s samples, but not the canine samples. To rule out introduction of sequencing artifacts given the low SARS-CoV-2 RNA concentrations in the canine samples, we repeated the sequencing workflow starting from the extracted RNA in a separate sequencing run with congruent results (Supplementary Fig. 1). Interestingly, we did not detect any other mutations than the ubiquitous D614G mutation in the spike gene of sequences from the dog samples.

**Fig. 4:**
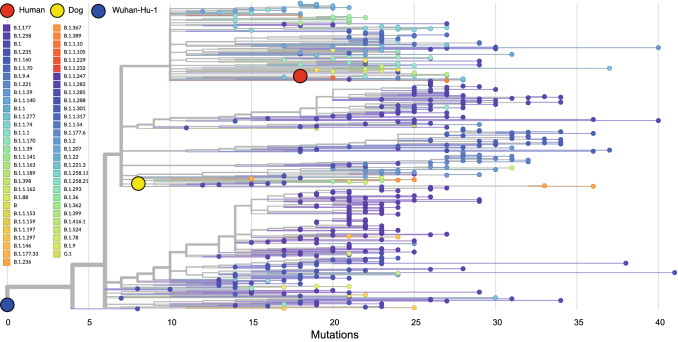
Phylogenetic analysis of canine and human samples. Maximum likelihood phylogenetic tree of whole genome SARS-CoV-2 sequences derived from the patient (red) and his dog (yellow) along with sequences sampled in Munich between September and December 2020 (*n* = 549) in relation to the reference genome Wuhan-Hu-1 (blue). A full list of sequences used in this analysis and their accession numbers is given in Supplementary Table 2. The *x*-axis represents the genetic distance as one nucleotide substitution in relation to the reference genome. The pangolin lineage assignments are indicated for each sequence using the color code shown in the insert in the upper left corner of the figure

We constructed a maximum-likelihood phylogeny using these sequences together with sequences from other clinical specimen (*n* = 549) collected in the larger Munich area between September and December 2020 (Fig. 4). Canine and human samples were classified as two separate SARS-CoV-2 lineages (B.1.1.29 and B.1.1.163, respectively) and are located on distinct branches of the phylogenetic tree illustrating their significant mutational differences.

## Discussion

So far, there is no proof of secondary zoonotic transmission of SARS-CoV-2 from dogs. Yet, due to the nature of coronaviruses, often originating from animal reservoirs and crossing the species barrier into humans, it seems that secondary zoonotic transmission from various species could be possible [17, 18]. SARS-CoV-2-infected minks and Syrian hamsters were reported to be prone to transmit the virus to humans [10, 11]. Furthermore, coronaviruses can adapt easily through mutations, increasing their resilience in new environments [19]. The present case illustrates the adaptability of SARS-CoV-2 and highlights the importance of viral genome sequencing for phylogenetic analysis.

In this infection cluster, the timeline of symptoms suggested secondary zoonotic transmission from a dog to a human. Chronologically, the patient’s symptoms began 6–10 days after the dog displayed respiratory signs, well within the typical incubation time of SARS-CoV-2 infections [20]. In addition, later serological testing confirmed seroconversion for antibodies against SARS-CoV-2 and therefore virus replication and infection in the dog, suggesting sufficient viral exposure had occurred at the moment of infection to trigger an adaptive immune response. Increased infectiousness was assumed due to the dog’s cough, being consistent with COVID-19 clinical signs and a possible mode of transmission via droplets or aerosols. Furthermore, all other family members tested negative for SARS-CoV-2 shortly after the patient’s symptoms commenced, rendering SARS-CoV-2 transmission from a family member to the patient very unlikely. Moreover, all family members developed symptoms and tested positive at a later point in time.

However, viral genome sequencing revealed two distinct virus variants for the patient and dog, respectively. In case transmission really had occurred from the dog to the patient, 14 de novo mutations would have emerged in the patient that were not present in the dog. Furthermore, the five mutations that were detected in the sequence of SARS-CoV-2 of the dog but not that of the patient would either have emerged in the dog after transmission to the patient or reverted to wild type in the patient. This scenario is so unlikely, that secondary zoonotic transmission can virtually be excluded. The same applies to the transmission from the patient to his dog. The similarity of the patient’s SARS-CoV-2 variant with other variants circulating in this geographical location at that time further supports separate community acquired infection (Fig. 4). The sequencing data therefore suggest that the preceding canine infection was coincidental and originated from another undefined source.

Our study has a few, but important limitations. Unfortunately, the respiratory samples of the other family members were tested in a different laboratory, so that no material was available for genomic analysis. Therefore, it cannot be ultimately excluded that these family members were infected directly by the dog. However, because the family father was the first symptomatic human case in this family and given that the other family members tested PCR-negative at the time when the father was tested positive, it is plausible that the other family members were infected directly by the father and not the dog. A limitation of this study was the low concentration of SARS-CoV-2 RNA in the canine samples. This may have resulted in sequencing artifacts due to contaminations in the sequencing pipeline. However, results of the three independent canine dog samples were congruent except for two mutations and reproducible as shown in the repetitive analyses.

In conclusion and in view of the current literature, SARS-CoV-2 dog-to-human transmission remains to be very unlikely. Nevertheless, secondary zoonotic transmission should be considered as a potential source of infection in particular for other species that are known to serve as a more permissive viral reservoir for SARS-CoV-2 such as minks and hamsters. We hope this case stresses the importance of viral genome sequencing for interspecies phylogenetic analysis of the SARS-CoV-2 pandemic when animal to human transmission is suspected.

## Supplementary Information

Below is the link to the electronic supplementary material.

Supplementary file1 Comparison of repeat sequencing runs using canine samples. The heatmap shows the frequencies of mutations in SARS-CoV-2 sequences obtained from the three canine samples in two separate sequencing runs in comparison to the reference genome (Wuhan-Hu-1). Genome positions with insufficient sequencing coverage (less than 20 reads at that position) are indicated as “NA”. Samples are labelled with sample number and date of sequencing run. (PDF 11 KB)

Supplementary file2 Sample characteristics. For each sample, viral load results, type of material, genome coverage, Pangolin lineage and GISAID accession IDs are indicated. (DOCX 14 KB)

Supplementary file3 Samples used for phylogenetic analysis. Sample IDs, collection date, GISAID accession numbers and Pangolin lineage assignment is indicated for samples collected between September and December 2020 in Munich (*n* = 549) and the reference genome Wuhan-Hu-1. (DOCX 43 KB)
