# Supplementary figures and images for: Secondary zoonotic dog-to-human transmission of SARS-CoV-2 suggested by timeline but refuted by viral genome sequencing
Source: Infection. 2022 Aug 20;51(1):253–9. doi: 10.1007/s15010-022-01902-y (PMC9392066; doi:10.1007/s15010-022-01902-y)

## Mutation heatmap

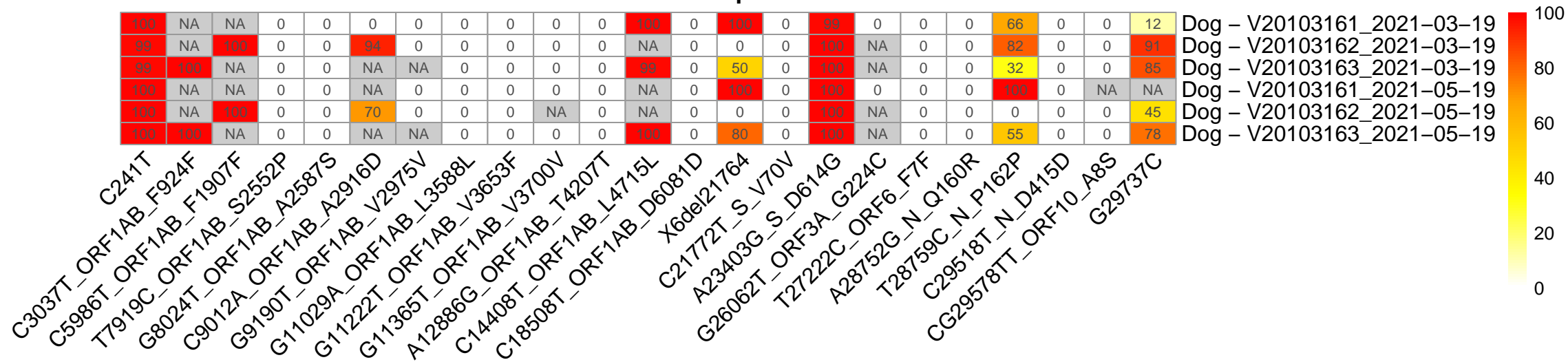

Supplement: Supplementary file 1 — Supplementary file1 Comparison of repeat sequencing runs using canine samples. The heatmap shows the frequencies of mutations in SARS-CoV-2 sequences obtained from the three canine samples in two separate sequencing runs in comparison to the reference genome (Wuhan-Hu-1). Genome positions with insufficient sequencing coverage (less than 20 reads at that position) are indicated as “NA”. Samples are labelled with sample number and date of sequencing run. (PDF 11 KB) [file 15010_2022_1902_MOESM1_ESM.pdf]
