# Supplementary material for: Secondary zoonotic dog-to-human transmission of SARS-CoV-2 suggested by timeline but refuted by viral genome sequencing
Source: Infection. 2022 Aug 20;51(1):253–9. doi: 10.1007/s15010-022-01902-y (PMC9392066; doi:10.1007/s15010-022-01902-y)
Supplement: Supplementary file 2 — Supplementary file2 Sample characteristics. For each sample, viral load results, type of material, genome coverage, Pangolin lineage and GISAID accession IDs are indicated. (DOCX 14 KB) [file 15010_2022_1902_MOESM2_ESM.docx]

| **Sample number** | **Ct** | **viral load (cp/ml)** | **collection date** | **age** | **sex** | **species** | **material** | **SARS-CoV-2 genome coverage** | **Pangolin lineage** | **GISAID EPI ISL** |
| --- | --- | --- | --- | --- | --- | --- | --- | --- | --- | --- |
| V20100859 | 26.6 | 766512 | 17/11/2020 | 44 | M | Human | nasopharyngeal swab | 99.88 | [B.1.1.163](https://outbreak.info/situation-reports?pango=B.1.1.163) | [1751110](https://gisaid.org/) |
| V20101672 | 23.7 | 15112714 | 19/11/2020 | 44 | M | Human | nasopharyngeal swab | 99.93 | [B.1.1.163](https://outbreak.info/situation-reports?pango=B.1.1.163) | [1751140](https://gisaid.org/) |
| V20102232 | 26.3 | 918059 | 22/11/2020 | 44 | M | Human | nasopharyngeal swab | NA |  |  |
| V20102808 | 33 | 27659 | 24/11/2020 | 44 | M | Human | nasopharyngeal swab | NA |  |  |
| V20103161 | 35.5 | 4945 | 24/11/2020 |  |  | Dog | pharyngeal swab | 84.79 | [B.1.1.29](https://outbreak.info/situation-reports?pango=B.1.1.29) | [1751194](https://gisaid.org/) |
| V20103162 | 33.5 | 19052 | 24/11/2020 |  |  | Dog | pharyngeal swab | 83.33 | [B.1.1.29](https://outbreak.info/situation-reports?pango=B.1.1.29) | [1751195](https://gisaid.org/) |
| V20103163 | 34 | 14044 | 24/11/2020 |  |  | Dog | pharyngeal swab | 77.68 | [B.1.1.29](https://outbreak.info/situation-reports?pango=B.1.1.29) | [1751196](https://gisaid.org/) |
