# Supplementary material for: Secondary zoonotic dog-to-human transmission of SARS-CoV-2 suggested by timeline but refuted by viral genome sequencing
Source: Infection. 2022 Aug 20;51(1):253–9. doi: 10.1007/s15010-022-01902-y (PMC9392066; doi:10.1007/s15010-022-01902-y)
Supplement: Supplementary file 3 — Supplementary file3 Samples used for phylogenetic analysis. Sample IDs, collection date, GISAID accession numbers and Pangolin lineage assignment is indicated for samples collected between September and December 2020 in Munich (n = 549) and the reference genome Wuhan-Hu-1. (DOCX 43 KB) [file 15010_2022_1902_MOESM3_ESM.docx]

| **Sample ID** | **Collection date** | **GISAID accession ID** | **Pangolin lineage** |
| --- | --- | --- | --- |
| **Wuhan/Hu-1/2019** | 2019-12-26 | EPI_ISL_402125 | B |
| **V20108464** | 2020-12-09 | EPI_ISL_1751330 | B.1.146 |
| **V2096160** | 2020-10-30 | EPI_ISL_1751718 | B.1.146 |
| **V20101653** | 2020-11-19 | EPI_ISL_1751138 | B.1 |
| **V2099938** | 2020-11-13 | EPI_ISL_1751804 | B.1 |
| **V20106473** | 2020-12-07 | EPI_ISL_1751304 | B.1 |
| **V2060851** | 2020-11-04 | EPI_ISL_1751515 | B.1.177 |
| **V20103162** | 2020-11-24 | EPI_ISL_1751195 | B.1.177 |
| **V2092474** | 2020-10-19 | EPI_ISL_1751651 | B.1.1.1 |
| **V2096121** | 2020-10-30 | EPI_ISL_2095073 | B.1.177 |
| **V2061402** | 2020-11-07 | EPI_ISL_1751522 | B.1 |
| **V20109217** | 2020-12-13 | EPI_ISL_1751358 | B.1.235 |
| **V20107253** | 2020-12-09 | EPI_ISL_1751325 | B.1.177 |
| **V2094988** | 2020-10-27 | EPI_ISL_1751680 | B.1 |
| **V20104043** | 2020-11-29 | EPI_ISL_1751222 | B.1.177 |
| **V2095094** | 2020-10-27 | EPI_ISL_1751683 | B.1 |
| **V20106139** | 2020-12-06 | EPI_ISL_1751282 | B.1.177 |
| **V20106873** | 2020-12-08 | EPI_ISL_1751312 | B.1.258 |
| **V20106506** | 2020-12-06 | EPI_ISL_1751305 | B.1 |
| **V20100199** | 2020-11-14 | EPI_ISL_1751077 | B.1 |
| **V2098044** | 2020-11-06 | EPI_ISL_2095061 | B.1.22 |
| **V20101621** | 2020-11-19 | EPI_ISL_1751135 | B.1.9 |
| **V2061408** | 2020-11-07 | EPI_ISL_1751523 | B.1 |
| **V2061134** | 2020-11-05 | EPI_ISL_1751518 | B.1 |
| **V2067398** | 2020-12-13 | EPI_ISL_1751581 | B.1.1.197 |
| **V20109795** | 2020-12-14 | EPI_ISL_1751378 | B.1.1.197 |
| **V2097485** | 2020-11-04 | EPI_ISL_1751753 | B.1.160 |
| **V2085766** | 2020-09-25 | EPI_ISL_1751613 | B.1.177 |
| **V2088551** | 2020-10-05 | EPI_ISL_1751627 | B.1.177 |
| **V2088990** | 2020-10-06 | EPI_ISL_1751633 | B.1 |
| **V2095821** | 2020-10-29 | EPI_ISL_1751706 | B.1.177 |
| **V2086356** | 2020-09-28 | EPI_ISL_1751615 | B.1.177 |
| **V2097801** | 2020-11-05 | EPI_ISL_1751761 | B.1.177 |
| **V20200226** | 2020-12-15 | EPI_ISL_1751496 | B.1.177 |
| **V2061378** | 2020-11-07 | EPI_ISL_1751520 | B.1 |
| **V20104085** | 2020-11-29 | EPI_ISL_1751224 | B.1 |
| **V2099360** | 2020-11-11 | EPI_ISL_1751784 | B.1 |
| **V20103401** | 2020-11-25 | EPI_ISL_1751201 | B.1.293 |
| **V20104142** | 2020-11-29 | EPI_ISL_1751228 | B.1.235 |
| **V20105498** | 2020-12-03 | EPI_ISL_1751261 | B.1.88 |
| **V20106233** | 2020-12-06 | EPI_ISL_1751295 | B.1.88 |
| **V20108467** | 2020-12-10 | EPI_ISL_1751331 | B.1.88 |
| **V20102791** | 2020-11-23 | EPI_ISL_1751176 | B.1.235 |
| **V20103866** | 2020-11-27 | EPI_ISL_1751216 | B.1.235 |
| **V20100592** | 2020-11-16 | EPI_ISL_1751100 | B.1.235 |
| **V20103121** | 2020-11-24 | EPI_ISL_1751191 | B.1.235 |
| **V20103108** | 2020-11-24 | EPI_ISL_1751189 | B.1 |
| **V20102337** | 2020-11-22 | EPI_ISL_1751163 | B.1.235 |
| **V2096637** | 2020-11-02 | EPI_ISL_1751733 | B.1.177 |
| **V2096749** | 2020-11-02 | EPI_ISL_1751740 | B.1.258 |
| **V2095527** | 2020-10-28 | EPI_ISL_2094557 | B.1.258.11 |
| **V2088406** | 2020-10-05 | EPI_ISL_1751626 | B.1 |
| **V2095589** | 2020-10-29 | EPI_ISL_1751696 | B.1.177 |
| **V2096862** | 2020-11-02 | EPI_ISL_1751742 | B.1.1 |
| **V20102573** | 2020-11-23 | EPI_ISL_1751167 | B.1.235 |
| **V20102781** | 2020-11-23 | EPI_ISL_1751174 | B.1 |
| **V20100409** | 2020-11-15 | EPI_ISL_1751093 | B.1.235 |
| **V20103698** | 2020-11-27 | EPI_ISL_1751214 | B.1.235 |
| **V20100061** | 2020-11-13 | EPI_ISL_1751071 | B.1.235 |
| **V20103979** | 2020-11-27 | EPI_ISL_1751218 | B.1.235 |
| **V20101551** | 2020-11-19 | EPI_ISL_1751130 | B.1.235 |
| **V20104112** | 2020-11-29 | EPI_ISL_1751227 | B.1.221 |
| **V20101949** | 2020-11-20 | EPI_ISL_1751151 | B.1.235 |
| **V20104144** | 2020-11-29 | EPI_ISL_1751229 | B.1.1.277 |
| **V20104038** | 2020-11-28 | EPI_ISL_1751221 | B.1.399 |
| **V2088663** | 2020-10-06 | EPI_ISL_1751629 | B.1.235 |
| **V20100090** | 2020-11-13 | EPI_ISL_1751073 | B.1.235 |
| **V20103709** | 2020-11-27 | EPI_ISL_1751215 | B.1.235 |
| **V20101542** | 2020-11-18 | EPI_ISL_1751129 | B.1.235 |
| **V20101649** | 2020-11-19 | EPI_ISL_1751137 | B.1.235 |
| **V20100353** | 2020-11-15 | EPI_ISL_1751085 | B.1.235 |
| **V20100794** | 2020-11-16 | EPI_ISL_1751107 | B.1 |
| **V20104109** | 2020-11-29 | EPI_ISL_1751226 | B.1.177 |
| **V20103090** | 2020-11-24 | EPI_ISL_1751186 | B.1.235 |
| **V20102506** | 2020-11-23 | EPI_ISL_1751165 | B.1.235 |
| **V20100092** | 2020-11-13 | EPI_ISL_1751074 | B.1.235 |
| **V20100760** | 2020-11-16 | EPI_ISL_1751105 | B.1.235 |
| **V20101304** | 2020-11-16 | EPI_ISL_1751123 | B.1.235 |
| **V20103115** | 2020-11-24 | EPI_ISL_1751190 | B.1.235 |
| **V20101665** | 2020-11-18 | EPI_ISL_1751139 | B.1.235 |
| **V20100362** | 2020-11-15 | EPI_ISL_1751089 | B.1.235 |
| **V20100699** | 2020-11-16 | EPI_ISL_1751103 | B.1 |
| **V20104762** | 2020-11-30 | EPI_ISL_1751244 | B.1 |
| **V20100211** | 2020-11-14 | EPI_ISL_1751078 | B.1.235 |
| **V20102941** | 2020-11-24 | EPI_ISL_1751181 | B.1.235 |
| **V20103610** | 2020-11-27 | EPI_ISL_1751210 | B.1.235 |
| **V20100340** | 2020-11-15 | EPI_ISL_1751083 | B.1.235 |
| **V20100993** | 2020-11-16 | EPI_ISL_1751114 | B.1.235 |
| **V20101475** | 2020-11-19 | EPI_ISL_1751127 | B.1.235 |
| **V20101939** | 2020-11-20 | EPI_ISL_1751150 | B.1.177 |
| **V20102063** | 2020-11-21 | EPI_ISL_1751155 | B.1 |
| **V20102783** | 2020-11-23 | EPI_ISL_1751175 | B.1.235 |
| **V20102838** | 2020-11-24 | EPI_ISL_1751179 | B.1.235 |
| **V20101715** | 2020-11-19 | EPI_ISL_1751143 | B.1.177 |
| **V20100186** | 2020-11-14 | EPI_ISL_1751076 | B.1.235 |
| **V20107197** | 2020-12-09 | EPI_ISL_1751324 | B.1.177 |
| **V20101705** | 2020-11-19 | EPI_ISL_1751142 | B.1.235 |
| **V20100375** | 2020-11-15 | EPI_ISL_1751092 | B.1.235 |
| **V20109245** | 2020-12-13 | EPI_ISL_1751360 | B.1.177 |
| **V2061091** | 2020-11-05 | EPI_ISL_1751516 | B.1.398 |
| **V2094980** | 2020-10-27 | EPI_ISL_1751679 | B.1 |
| **V2095680** | 2020-10-29 | EPI_ISL_1751700 | B.1.177 |
| **V2060660** | 2020-11-03 | EPI_ISL_1751508 | B.1.177 |
| **V2087321** | 2020-10-01 | EPI_ISL_1751619 | B.1.177 |
| **V2095282** | 2020-10-28 | EPI_ISL_1751689 | B.1.177 |
| **V2096463** | 2020-11-01 | EPI_ISL_1751728 | B.1.177 |
| **V20106094** | 2020-12-06 | EPI_ISL_1751279 | B.1.177 |
| **V2064706** | 2020-11-26 | EPI_ISL_1751559 | B.1.177 |
| **V2064506** | 2020-11-24 | EPI_ISL_1751554 | B.1.177 |
| **V2096107** | 2020-10-30 | EPI_ISL_1751716 | B.1.177 |
| **V2094749** | 2020-10-26 | EPI_ISL_1751673 | B.1.177 |
| **V2095597** | 2020-10-28 | EPI_ISL_1751697 | B.1.177 |
| **V20101857** | 2020-11-20 | EPI_ISL_1751146 | B.1.177 |
| **V20104107** | 2020-11-29 | EPI_ISL_1751225 | B.1.177 |
| **V20103403** | 2020-11-26 | EPI_ISL_1751202 | B.1.177 |
| **V20101859** | 2020-11-20 | EPI_ISL_1751147 | B.1.177 |
| **V20100481** | 2020-11-15 | EPI_ISL_1751097 | B.1.177 |
| **V20109727** | 2020-12-14 | EPI_ISL_1751371 | B.1.177.6 |
| **V2067415** | 2020-12-14 | EPI_ISL_1751582 | B.1.177 |
| **V2095498** | 2020-10-28 | EPI_ISL_1751695 | B.1.177 |
| **V2095793** | 2020-10-29 | EPI_ISL_1751703 | B.1.177 |
| **V2099636** | 2020-11-12 | EPI_ISL_1751795 | B.1.177 |
| **V20106193** | 2020-12-06 | EPI_ISL_1751293 | B.1.177 |
| **V20104185** | 2020-11-29 | EPI_ISL_1751230 | B.1.177 |
| **V2097115** | 2020-11-03 | EPI_ISL_1751747 | B.1.177 |
| **V2066300** | 2020-12-07 | EPI_ISL_1751570 | B.1.177 |
| **V20109782** | 2020-12-15 | EPI_ISL_1751377 | B.1.177 |
| **V20107149** | 2020-12-09 | EPI_ISL_1751322 | B.1.177 |
| **V2096245** | 2020-10-31 | EPI_ISL_1751720 | B.1.177 |
| **V2096725** | 2020-11-02 | EPI_ISL_1751739 | B.1.177.33 |
| **V2096218** | 2020-10-30 | EPI_ISL_1751719 | B.1.177.33 |
| **V2099617** | 2020-11-12 | EPI_ISL_1751793 | B.1.177 |
| **V2094092** | 2020-10-24 | EPI_ISL_1751664 | B.1.177 |
| **V2098385** | 2020-11-08 | EPI_ISL_1751770 | B.1.177 |
| **V20109621** | 2020-12-14 | EPI_ISL_1751367 | B.1.177 |
| **V2060486** | 2020-11-02 | EPI_ISL_1751505 | B.1.177 |
| **V2106687** | 2020-12-02 | EPI_ISL_1752026 | B.1.177 |
| **V2106685** | 2020-11-11 | EPI_ISL_1752024 | B.1.177 |
| **V2106683** | 2020-11-04 | EPI_ISL_1752022 | B.1.177 |
| **V2060711** | 2020-11-03 | EPI_ISL_1751511 | B.1.177 |
| **V20100795** | 2020-11-16 | EPI_ISL_1751108 | B.1.177 |
| **V2066536** | 2020-12-08 | EPI_ISL_1751571 | B.1.177 |
| **V20106179** | 2020-12-06 | EPI_ISL_1751291 | B.1.177 |
| **V20101176** | 2020-11-17 | EPI_ISL_1751118 | B.1.177 |
| **V2063939** | 2020-11-21 | EPI_ISL_1751546 | B.1.177 |
| **V2087302** | 2020-10-01 | EPI_ISL_2095256 | B.1.177 |
| **V20109761** | 2020-12-14 | EPI_ISL_1751374 | B.1.177 |
| **V20109809** | 2020-12-14 | EPI_ISL_1751380 | B.1.177 |
| **V20106961** | 2020-12-08 | EPI_ISL_1751315 | B.1.177 |
| **V20106753** | 2020-12-07 | EPI_ISL_1751309 | B.1.177 |
| **V20106722** | 2020-12-07 | EPI_ISL_1751308 | B.1.177 |
| **V20108478** | 2020-12-09 | EPI_ISL_1751332 | B.1.177 |
| **V20106414** | 2020-12-07 | EPI_ISL_1751303 | B.1.177 |
| **V20106413** | 2020-12-07 | EPI_ISL_1751302 | B.1.177 |
| **V20109008** | 2020-12-11 | EPI_ISL_1751351 | B.1.177 |
| **V20106152** | 2020-12-06 | EPI_ISL_1751286 | B.1.177 |
| **V20103620** | 2020-11-26 | EPI_ISL_1751211 | B.1.177 |
| **V20106333** | 2020-12-07 | EPI_ISL_1751299 | B.1.177 |
| **V2066187** | 2020-12-05 | EPI_ISL_1751565 | B.1.177 |
| **V20108760** | 2020-12-10 | EPI_ISL_1751342 | B.1.177 |
| **V20106141** | 2020-12-06 | EPI_ISL_1751284 | B.1.177 |
| **V20106963** | 2020-12-08 | EPI_ISL_1751316 | B.1.177 |
| **V20106335** | 2020-12-07 | EPI_ISL_1751300 | B.1.177 |
| **V20106159** | 2020-12-06 | EPI_ISL_1751287 | B.1.177 |
| **V20106408** | 2020-12-07 | EPI_ISL_1751301 | B.1.177 |
| **V20106326** | 2020-12-07 | EPI_ISL_1751298 | B.1.177 |
| **V20106161** | 2020-12-06 | EPI_ISL_1751289 | B.1.177 |
| **V20106142** | 2020-12-06 | EPI_ISL_1751285 | B.1.177 |
| **V20106140** | 2020-12-06 | EPI_ISL_1751283 | B.1.177 |
| **V20106160** | 2020-12-06 | EPI_ISL_1751288 | B.1.177 |
| **V2067382** | 2020-12-13 | EPI_ISL_1751580 | B.1.177 |
| **V2067864** | 2020-12-15 | EPI_ISL_1751584 | B.1.177 |
| **V2096524** | 2020-11-01 | EPI_ISL_1751732 | B.1.177 |
| **V20105864** | 2020-12-04 | EPI_ISL_1751269 | B.1.177 |
| **V2060692** | 2020-11-03 | EPI_ISL_1751509 | B.1.177 |
| **V2098689** | 2020-11-05 | EPI_ISL_1751775 | B.1.177 |
| **V2067287** | 2020-12-12 | EPI_ISL_1751577 | B.1.177 |
| **V20102075** | 2020-11-20 | EPI_ISL_1751156 | B.1.177 |
| **V2064552** | 2020-11-25 | EPI_ISL_1751555 | B.1.177 |
| **V20100358** | 2020-11-15 | EPI_ISL_1751087 | B.1.177 |
| **V20103670** | 2020-11-26 | EPI_ISL_1751212 | B.1.177 |
| **V20108789** | 2020-12-11 | EPI_ISL_1751344 | B.1.177 |
| **V20200157** | 2020-12-08 | EPI_ISL_1751493 | B.1.177 |
| **V2065465** | 2020-12-01 | EPI_ISL_1751564 | B.1.177 |
| **V2093864** | 2020-10-23 | EPI_ISL_1751661 | B.1.177 |
| **V2092175** | 2020-10-18 | EPI_ISL_1751649 | B.1.177 |
| **V2094363** | 2020-10-25 | EPI_ISL_1751667 | B.1.177 |
| **V20100663** | 2020-11-16 | EPI_ISL_1751102 | B.1.177 |
| **V2065054** | 2020-11-28 | EPI_ISL_1751562 | B.1.177 |
| **V20109125** | 2020-12-12 | EPI_ISL_2095047 | B.1.177 |
| **V20105984** | 2020-12-06 | EPI_ISL_1751273 | B.1.177 |
| **V20105850** | 2020-12-05 | EPI_ISL_1751268 | B.1.177 |
| **V2063796** | 2020-11-20 | EPI_ISL_1751542 | B.1.177 |
| **V2084809** | 2020-09-21 | EPI_ISL_1751610 | B.1.177 |
| **V2099722** | 2020-11-12 | EPI_ISL_1751800 | B.1.177 |
| **V2099656** | 2020-11-12 | EPI_ISL_1751796 | B.1.177 |
| **V2056301** | 2020-10-06 | EPI_ISL_1751504 | B.1.177 |
| **V20104034** | 2020-11-28 | EPI_ISL_1751220 | B.1.177 |
| **V20104556** | 2020-11-30 | EPI_ISL_1751238 | B.1.177 |
| **V2095495** | 2020-10-28 | EPI_ISL_1751694 | B.1.177 |
| **V2095624** | 2020-10-29 | EPI_ISL_1751698 | B.1.177 |
| **V2096385** | 2020-11-01 | EPI_ISL_1751726 | B.1.177 |
| **V2095382** | 2020-10-28 | EPI_ISL_1751692 | B.1.177 |
| **V20200176** | 2020-12-10 | EPI_ISL_1751495 | B.1.177 |
| **V2088925** | 2020-10-06 | EPI_ISL_1751632 | B.1.177 |
| **V2089471** | 2020-10-08 | EPI_ISL_1751635 | B.1.177 |
| **V2099853** | 2020-11-12 | EPI_ISL_1751802 | B.1.177 |
| **V20100866** | 2020-11-17 | EPI_ISL_1751112 | B.1.177 |
| **V20102217** | 2020-11-22 | EPI_ISL_1751158 | B.1.177 |
| **V20100906** | 2020-11-17 | EPI_ISL_1751113 | B.1.177 |
| **V2062712** | 2020-11-15 | EPI_ISL_1751533 | B.1.177 |
| **V2099794** | 2020-11-12 | EPI_ISL_1751801 | B.1.177 |
| **V20106310** | 2020-12-07 | EPI_ISL_1751296 | B.1.177 |
| **V20101403** | 2020-11-18 | EPI_ISL_1751126 | B.1.177 |
| **V2066575** | 2020-12-08 | EPI_ISL_1751573 | B.1.177 |
| **V20109476** | 2020-12-14 | EPI_ISL_1751366 | B.1.177 |
| **V20106870** | 2020-12-08 | EPI_ISL_1751310 | B.1.177 |
| **V20106232** | 2020-12-06 | EPI_ISL_1751294 | B.1.177 |
| **V2067159** | 2020-12-11 | EPI_ISL_1751576 | B.1.177 |
| **V2099342** | 2020-11-11 | EPI_ISL_1751783 | B.1 |
| **V2096673** | 2020-11-02 | EPI_ISL_1751738 | B.1.367 |
| **SWAB11** | 2020-10-28 | EPI_ISL_1751064 | B.1.367 |
| **V2094393** | 2020-10-25 | EPI_ISL_1751668 | B.1 |
| **V2094773** | 2020-10-26 | EPI_ISL_1751674 | B.1 |
| **V20103575** | 2020-11-26 | EPI_ISL_1751208 | B.1.258 |
| **V20103161** | 2020-11-24 | EPI_ISL_1751194 | B.1 |
| **V20100215** | 2020-11-14 | EPI_ISL_1751079 | B.1 |
| **V20100323** | 2020-11-15 | EPI_ISL_1751082 | B.1.398 |
| **V20100312** | 2020-11-15 | EPI_ISL_1751081 | B.1.398 |
| **V2095167** | 2020-10-27 | EPI_ISL_1751685 | B.1.36 |
| **V2085799** | 2020-09-25 | EPI_ISL_1751614 | B.1.362 |
| **V2061416** | 2020-11-07 | EPI_ISL_1751525 | B.1.389 |
| **V2095981** | 2020-10-30 | EPI_ISL_1751711 | B.1.389 |
| **V20101987** | 2020-11-20 | EPI_ISL_1751152 | B.1.236 |
| **V2087752** | 2020-10-02 | EPI_ISL_1751620 | B.1.236 |
| **V20103163** | 2020-11-24 | EPI_ISL_1751196 | B.1 |
| **V20102808** | 2020-11-24 | EPI_ISL_1751178 | B.1.398 |
| **V2096338** | 2020-10-31 | EPI_ISL_1751721 | B.1.2 |
| **V20108645** | 2020-12-10 | EPI_ISL_1751338 | B.1.235 |
| **V20102230** | 2020-11-22 | EPI_ISL_1751160 | B.1.235 |
| **V2091851** | 2020-10-16 | EPI_ISL_1751646 | B.1.221 |
| **V20101562** | 2020-11-19 | EPI_ISL_1751131 | B.1.221 |
| **V20103138** | 2020-11-25 | EPI_ISL_1751193 | B.1.177 |
| **V20107035** | 2020-12-08 | EPI_ISL_1751320 | B.1.221 |
| **V20108646** | 2020-12-10 | EPI_ISL_1751339 | B.1.221 |
| **V20100850** | 2020-11-17 | EPI_ISL_1751109 | B.1.221 |
| **V2095259** | 2020-10-28 | EPI_ISL_1751688 | B.1.221 |
| **V2062698** | 2020-11-14 | EPI_ISL_1751532 | B.1.221 |
| **V2065412** | 2020-11-30 | EPI_ISL_1751563 | B.1.221.3 |
| **V20102792** | 2020-11-23 | EPI_ISL_1751177 | B.1.221 |
| **V2095699** | 2020-10-29 | EPI_ISL_1751701 | B.1.221 |
| **V2063717** | 2020-11-20 | EPI_ISL_1751541 | B.1.221 |
| **V2096086** | 2020-10-30 | EPI_ISL_1751715 | B.1.221 |
| **V20108516** | 2020-12-10 | EPI_ISL_1751334 | B.1.524 |
| **V2064876** | 2020-11-26 | EPI_ISL_1751560 | B.1.221 |
| **V20108970** | 2020-12-11 | EPI_ISL_1751349 | B.1.221 |
| **V2095189** | 2020-10-27 | EPI_ISL_1751686 | B.1 |
| **V20101197** | 2020-11-17 | EPI_ISL_1751119 | B.1 |
| **V20102744** | 2020-11-23 | EPI_ISL_1751169 | B.1.177 |
| **V2062656** | 2020-11-13 | EPI_ISL_1751530 | B.1 |
| **V2094944** | 2020-10-26 | EPI_ISL_1751678 | B.1.9.4 |
| **V2096519** | 2020-11-01 | EPI_ISL_1751731 | B.1.177 |
| **V20103190** | 2020-11-25 | EPI_ISL_1751197 | B.1.9.4 |
| **V2067124** | 2020-12-10 | EPI_ISL_1751574 | B.1.9.4 |
| **V20106103** | 2020-12-06 | EPI_ISL_1751281 | B.1.9.4 |
| **V2090762** | 2020-10-13 | EPI_ISL_1751637 | B.1.9.4 |
| **V2094205** | 2020-10-24 | EPI_ISL_1751666 | B.1.9.4 |
| **V20101500** | 2020-11-18 | EPI_ISL_1751128 | B.1.9.4 |
| **V20101055** | 2020-11-17 | EPI_ISL_1751117 | B.1.9.4 |
| **V20101054** | 2020-11-17 | EPI_ISL_1751116 | B.1.9.4 |
| **V20108958** | 2020-12-11 | EPI_ISL_1751348 | B.1.9.4 |
| **V20103475** | 2020-11-26 | EPI_ISL_1751204 | B.1.9.4 |
| **V2095095** | 2020-10-27 | EPI_ISL_1751684 | B.1.9.4 |
| **V20102751** | 2020-11-23 | EPI_ISL_1751171 | B.1.9.4 |
| **V2099236** | 2020-11-10 | EPI_ISL_1751780 | B.1.9.4 |
| **V2096001** | 2020-10-30 | EPI_ISL_1751712 | B.1.9.4 |
| **V20101369** | 2020-11-18 | EPI_ISL_1751125 | B.1.9.4 |
| **V2097012** | 2020-11-02 | EPI_ISL_1751745 | B.1.9.4 |
| **V2097810** | 2020-11-05 | EPI_ISL_1751762 | B.1.9.4 |
| **V2098926** | 2020-11-09 | EPI_ISL_1751777 | B.1.9.4 |
| **V2064340** | 2020-11-24 | EPI_ISL_1751552 | B.1.9.4 |
| **V20100065** | 2020-11-13 | EPI_ISL_1751072 | B.1.9.4 |
| **V2064590** | 2020-11-25 | EPI_ISL_1751557 | B.1.160 |
| **V2063922** | 2020-11-21 | EPI_ISL_1751544 | B.1.160 |
| **V20103077** | 2020-11-24 | EPI_ISL_1751184 | B.1 |
| **V20105093** | 2020-12-02 | EPI_ISL_1751253 | B.1.160 |
| **V2062492** | 2020-11-12 | EPI_ISL_1751526 | B.1 |
| **V2063261** | 2020-11-17 | EPI_ISL_1751536 | B.1.177 |
| **V2064569** | 2020-11-25 | EPI_ISL_1751556 | B.1.177 |
| **V2063894** | 2020-11-20 | EPI_ISL_1751543 | B.1.221 |
| **V2097578** | 2020-11-04 | EPI_ISL_1751755 | B.1.160 |
| **V20101693** | 2020-11-19 | EPI_ISL_1751141 | B.1.160 |
| **V20101790** | 2020-11-19 | EPI_ISL_1751144 | B.1.160 |
| **V20103072** | 2020-11-24 | EPI_ISL_1751183 | B.1.160 |
| **V20104942** | 2020-12-01 | EPI_ISL_1751248 | B.1.160 |
| **V2096376** | 2020-10-31 | EPI_ISL_1751725 | B.1 |
| **V20108705** | 2020-12-10 | EPI_ISL_2094562 | B.1.160 |
| **V2097206** | 2020-11-03 | EPI_ISL_1751750 | B.1.160 |
| **V2061375** | 2020-11-07 | EPI_ISL_1751519 | B.1.160 |
| **V20100372** | 2020-11-15 | EPI_ISL_1751091 | B.1.160 |
| **V20109772** | 2020-12-14 | EPI_ISL_1751376 | B.1.160 |
| **V20105553** | 2020-12-03 | EPI_ISL_1751262 | B.1.160 |
| **V2095830** | 2020-10-29 | EPI_ISL_1751707 | B.1.160 |
| **V2093555** | 2020-10-22 | EPI_ISL_1751659 | B.1.160 |
| **V20107050** | 2020-12-08 | EPI_ISL_1751321 | B.1.160 |
| **V20110020** | 2020-12-15 | EPI_ISL_1751381 | B.1.160 |
| **V2066203** | 2020-12-06 | EPI_ISL_1751566 | B.1.160 |
| **V2066570** | 2020-12-08 | EPI_ISL_1751572 | B.1.160 |
| **V20105126** | 2020-12-02 | EPI_ISL_1751254 | B.1.160 |
| **V20106874** | 2020-12-08 | EPI_ISL_1751313 | B.1.160 |
| **V20105354** | 2020-12-03 | EPI_ISL_1751260 | B.1.160 |
| **V2092503** | 2020-10-20 | EPI_ISL_1751652 | B.1.160 |
| **V2094746** | 2020-10-26 | EPI_ISL_1751672 | B.1.160 |
| **V20108507** | 2020-12-09 | EPI_ISL_1751333 | B.1.160 |
| **V20104209** | 2020-11-29 | EPI_ISL_1751231 | B.1.160 |
| **V20103378** | 2020-11-25 | EPI_ISL_1751199 | B.1.160 |
| **V2097785** | 2020-11-05 | EPI_ISL_1751760 | B.1.160 |
| **V20105866** | 2020-12-04 | EPI_ISL_1751271 | B.1.160 |
| **V20105865** | 2020-12-04 | EPI_ISL_1751270 | B.1.160 |
| **V2099571** | 2020-11-11 | EPI_ISL_1751792 | B.1.160 |
| **V2066274** | 2020-12-07 | EPI_ISL_1751567 | B.1.160 |
| **V2096759** | 2020-11-02 | EPI_ISL_1751741 | B.1.160 |
| **V2061276** | 2020-11-06 | EPI_ISL_2095183 | B.1.160 |
| **V2062557** | 2020-11-13 | EPI_ISL_1751528 | B.1.160 |
| **V2097295** | 2020-11-03 | EPI_ISL_1751751 | B.1.416.1 |
| **V20101266** | 2020-11-18 | EPI_ISL_1751122 | B.1 |
| **V20104329** | 2020-11-30 | EPI_ISL_1751233 | B.1.258 |
| **V20103137** | 2020-11-25 | EPI_ISL_1751192 | B.1 |
| **V20104530** | 2020-11-30 | EPI_ISL_1751237 | B.1.258 |
| **V20108531** | 2020-12-10 | EPI_ISL_1751337 | B.1.258 |
| **V2062673** | 2020-11-14 | EPI_ISL_1751531 | G.1 |
| **V2066292** | 2020-12-07 | EPI_ISL_1751569 | B.1.221 |
| **V2064063** | 2020-11-23 | EPI_ISL_1751549 | B.1.258 |
| **V20104588** | 2020-11-30 | EPI_ISL_1751240 | B.1.258 |
| **V20102212** | 2020-11-22 | EPI_ISL_1751157 | B.1.258 |
| **V20108722** | 2020-12-10 | EPI_ISL_1751341 | B.1.258 |
| **V20102530** | 2020-11-23 | EPI_ISL_1751166 | B.1.258 |
| **V20102006** | 2020-11-20 | EPI_ISL_1751154 | B.1.235 |
| **V2091504** | 2020-10-15 | EPI_ISL_1751639 | B.1 |
| **V20106314** | 2020-12-07 | EPI_ISL_1751297 | B.1.258 |
| **V2096005** | 2020-10-30 | EPI_ISL_1751713 | B.1.177 |
| **V20102780** | 2020-11-23 | EPI_ISL_1751173 | B.1.258 |
| **V20101020** | 2020-11-17 | EPI_ISL_1751115 | B.1.258 |
| **V2095356** | 2020-10-28 | EPI_ISL_1751691 | B.1.258 |
| **V2099619** | 2020-11-12 | EPI_ISL_1751794 | B.1.258 |
| **V2097202** | 2020-11-03 | EPI_ISL_1751749 | B.1.258 |
| **V20200169** | 2020-12-09 | EPI_ISL_1751494 | B.1.258 |
| **V2067183** | 2020-12-11 | EPI_ISL_2095197 | B.1.258 |
| **V2067138** | 2020-12-11 | EPI_ISL_2094633 | B.1.258 |
| **V2063900** | 2020-11-20 | EPI_ISL_2095192 | B.1.258 |
| **V2096423** | 2020-10-31 | EPI_ISL_2095213 | B.1.258 |
| **V2094001** | 2020-10-23 | EPI_ISL_1751663 | B.1.258 |
| **V2091817** | 2020-10-17 | EPI_ISL_1751645 | B.1.258 |
| **V2090056** | 2020-10-11 | EPI_ISL_2095209 | B.1.258 |
| **V2063055** | 2020-11-16 | EPI_ISL_2095191 | B.1.258 |
| **V2067022** | 2020-12-10 | EPI_ISL_2095196 | B.1.258 |
| **V20100901** | 2020-11-17 | EPI_ISL_2094619 | B.1.258 |
| **V2066718** | 2020-12-08 | EPI_ISL_2095195 | B.1.258 |
| **V20101247** | 2020-11-17 | EPI_ISL_2095199 | B.1.258 |
| **V20102578** | 2020-11-23 | EPI_ISL_1751168 | B.1.258 |
| **V2063545** | 2020-11-19 | EPI_ISL_1751537 | B.1.258 |
| **V2064316** | 2020-11-24 | EPI_ISL_1751550 | B.1.258 |
| **V2064702** | 2020-11-26 | EPI_ISL_2095194 | B.1.258.21 |
| **V20102218** | 2020-11-22 | EPI_ISL_1751159 | B.1.258 |
| **V20100580** | 2020-11-16 | EPI_ISL_1751099 | B.1.258 |
| **V2098441** | 2020-11-08 | EPI_ISL_2095214 | B.1.258 |
| **V2066731** | 2020-12-09 | EPI_ISL_2094618 | B.1.258 |
| **V20109014** | 2020-12-11 | EPI_ISL_2095205 | B.1.258 |
| **V20108625** | 2020-12-10 | EPI_ISL_2095204 | B.1.258 |
| **V20105597** | 2020-12-03 | EPI_ISL_1751263 | B.1.258 |
| **V20104255** | 2020-11-30 | EPI_ISL_2095202 | B.1.258 |
| **V20104590** | 2020-11-30 | EPI_ISL_2095203 | B.1.258 |
| **V20200100** | 2020-12-03 | EPI_ISL_2095207 | B.1.258 |
| **V20104048** | 2020-11-28 | EPI_ISL_2095201 | B.1.258 |
| **V20107029** | 2020-12-08 | EPI_ISL_2094547 | B.1.258 |
| **V20106717** | 2020-12-07 | EPI_ISL_2094524 | B.1.258 |
| **V20102551** | 2020-11-23 | EPI_ISL_2094531 | B.1.258 |
| **V2095151** | 2020-10-27 | EPI_ISL_2095211 | B.1.258 |
| **V20103397** | 2020-11-26 | EPI_ISL_1751200 | B.1.258 |
| **V20108811** | 2020-12-11 | EPI_ISL_2094596 | B.1.258 |
| **V20106089** | 2020-12-06 | EPI_ISL_2094582 | B.1.258 |
| **V20106472** | 2020-12-06 | EPI_ISL_2094598 | B.1.258 |
| **V2092026** | 2020-10-17 | EPI_ISL_2095210 | B.1.258 |
| **V20102137** | 2020-11-21 | EPI_ISL_2094579 | B.1.258 |
| **V2089580** | 2020-10-08 | EPI_ISL_2095208 | B.1.258 |
| **V2088397** | 2020-10-05 | EPI_ISL_1751625 | B.1.258 |
| **V20109090** | 2020-12-11 | EPI_ISL_1751354 | B.1.258 |
| **V2064074** | 2020-11-23 | EPI_ISL_2095193 | B.1.258 |
| **V20104853** | 2020-12-01 | EPI_ISL_852603 | B.1.258 |
| **V2106688** | 2020-11-26 | EPI_ISL_2095254 | B.1.258 |
| **V20104327** | 2020-11-30 | EPI_ISL_852602 | B.1.258 |
| **V20103484** | 2020-11-26 | EPI_ISL_852601 | B.1.258 |
| **V2106680** | 2020-10-20 | EPI_ISL_1752020 | B.1.258 |
| **V2095236** | 2020-10-28 | EPI_ISL_2095212 | B.1.258 |
| **V20104465** | 2020-11-30 | EPI_ISL_1751236 | B.1.258 |
| **V20102562** | 2020-11-23 | EPI_ISL_2095239 | B.1.258 |
| **V20101617** | 2020-11-19 | EPI_ISL_1751134 | B.1.258 |
| **V2106678** | 2020-10-15 | EPI_ISL_2094571 | B.1.258 |
| **V2094093** | 2020-10-24 | EPI_ISL_852575 | B.1.258 |
| **V2099887** | 2020-11-13 | EPI_ISL_1751803 | B.1.258 |
| **V2106681** | 2020-10-22 | EPI_ISL_1752021 | B.1.258 |
| **V2106677** | 2020-10-20 | EPI_ISL_2094611 | B.1.258 |
| **V2092772** | 2020-10-20 | EPI_ISL_852599 | B.1.258 |
| **V2106674** | 2020-10-20 | EPI_ISL_1752018 | B.1.258 |
| **V2106676** | 2020-10-21 | EPI_ISL_2094555 | B.1.258 |
| **V2106689** | 2020-10-22 | EPI_ISL_2095255 | B.1.258 |
| **V2106679** | 2020-10-23 | EPI_ISL_2094537 | B.1.258 |
| **V2106682** | 2020-10-20 | EPI_ISL_2094533 | B.1.258 |
| **V2106673** | 2020-10-26 | EPI_ISL_2094545 | B.1.258 |
| **V2094061** | 2020-10-23 | EPI_ISL_852600 | B.1.258 |
| **V20104720** | 2020-11-30 | EPI_ISL_1751242 | B.1.1.10 |
| **V20108950** | 2020-12-11 | EPI_ISL_1751347 | B.1.1.29 |
| **V2106675** | 2020-11-05 | EPI_ISL_1752019 | B.1.1.247 |
| **SWAB13** | 2020-10-30 | EPI_ISL_1751066 | B.1.1 |
| **V2088256** | 2020-10-05 | EPI_ISL_1751624 | B.1.1.54 |
| **V2060494** | 2020-11-02 | EPI_ISL_1751506 | B.1.1.105 |
| **V2095923** | 2020-10-30 | EPI_ISL_1751709 | B.1.1 |
| **V2082641** | 2020-09-10 | EPI_ISL_1751607 | B.1.1.74 |
| **V2083593** | 2020-09-17 | EPI_ISL_1751608 | B.1.1.74 |
| **V2088212** | 2020-10-05 | EPI_ISL_1751623 | B.1.1.29 |
| **V2085389** | 2020-09-24 | EPI_ISL_1751611 | B.1.1.74 |
| **V20105187** | 2020-12-02 | EPI_ISL_1751255 | B.1.1.163 |
| **V20100859** | 2020-11-17 | EPI_ISL_1751110 | B.1.1.163 |
| **V20101672** | 2020-11-19 | EPI_ISL_1751140 | B.1.1.163 |
| **V20102232** | 2020-11-22 | EPI_ISL_1751161 | B.1.1.163 |
| **V2060715** | 2020-11-03 | EPI_ISL_1751512 | B.1.1.282 |
| **V2092028** | 2020-10-17 | EPI_ISL_1751647 | B.1.1.162 |
| **V20100371** | 2020-11-15 | EPI_ISL_1751090 | B.1.1.162 |
| **V2094791** | 2020-10-26 | EPI_ISL_1751675 | B.1.1.162 |
| **V2099428** | 2020-11-11 | EPI_ISL_1751786 | B.1.1.159 |
| **V2106684** | 2020-10-22 | EPI_ISL_1752023 | B.1.1.159 |
| **V2060496** | 2020-11-02 | EPI_ISL_1751507 | B.1.1.189 |
| **V2096355** | 2020-10-31 | EPI_ISL_1751723 | B.1.1 |
| **V20106177** | 2020-12-06 | EPI_ISL_1751290 | B.1.1.189 |
| **V20105288** | 2020-12-02 | EPI_ISL_1751258 | B.1.1.189 |
| **V20101930** | 2020-11-20 | EPI_ISL_1751149 | B.1.1.189 |
| **V2091701** | 2020-10-16 | EPI_ISL_1751644 | B.1.1.1 |
| **V20105300** | 2020-12-02 | EPI_ISL_1751259 | B.1.1.1 |
| **V2067358** | 2020-12-12 | EPI_ISL_1751578 | B.1 |
| **V2085476** | 2020-09-24 | EPI_ISL_1751612 | B.1.1.1 |
| **V20107301** | 2020-12-09 | EPI_ISL_1751328 | B.1.1.285 |
| **V20109631** | 2020-12-14 | EPI_ISL_1751368 | B.1.1.1 |
| **V20102754** | 2020-11-23 | EPI_ISL_1751172 | B.1.1.1 |
| **V20100360** | 2020-11-15 | EPI_ISL_1751088 | B.1.1.153 |
| **V2092049** | 2020-10-18 | EPI_ISL_1751648 | B.1 |
| **V20103676** | 2020-11-26 | EPI_ISL_1751213 | B.1 |
| **SWAB09** | 2020-10-22 | EPI_ISL_1751063 | B.1.1.153 |
| **V2094999** | 2020-10-27 | EPI_ISL_1751681 | B.1.1.170 |
| **V20103496** | 2020-11-26 | EPI_ISL_1751205 | B.1.1.170 |
| **V2098272** | 2020-11-07 | EPI_ISL_1751769 | B.1.1.170 |
| **SWAB05** | 2020-10-12 | EPI_ISL_1751061 | B.1 |
| **V20109169** | 2020-12-12 | EPI_ISL_1751355 | B.1.1.170 |
| **V20100343** | 2020-11-15 | EPI_ISL_1751084 | B.1.1.170 |
| **V2096400** | 2020-10-31 | EPI_ISL_1751727 | B.1.1.170 |
| **V20100279** | 2020-11-14 | EPI_ISL_1751080 | B.1.1.170 |
| **V2060753** | 2020-11-04 | EPI_ISL_1751514 | B.1.1 |
| **V20103909** | 2020-11-27 | EPI_ISL_1751217 | B.1.1.29 |
| **V20104784** | 2020-11-30 | EPI_ISL_1751245 | B.1.1.29 |
| **V20103099** | 2020-11-25 | EPI_ISL_1751187 | B.1 |
| **V2093019** | 2020-10-20 | EPI_ISL_1751654 | B.1.1.301 |
| **V20100477** | 2020-11-15 | EPI_ISL_1751095 | B.1.1.29 |
| **V2089326** | 2020-10-08 | EPI_ISL_1751634 | B.1.1.229 |
| **V20106978** | 2020-12-08 | EPI_ISL_1751317 | B.1.1.1 |
| **V20106650** | 2020-12-07 | EPI_ISL_1751306 | B.1.1.29 |
| **V20106985** | 2020-12-08 | EPI_ISL_1751318 | B.1.207 |
| **V20106651** | 2020-12-07 | EPI_ISL_1751307 | B.1.78 |
| **V20106939** | 2020-12-08 | EPI_ISL_1751314 | B.1.177 |
| **V2060710** | 2020-11-03 | EPI_ISL_1751510 | B.1 |
| **V2094793** | 2020-10-26 | EPI_ISL_1751676 | B.1 |
| **V2094480** | 2020-10-24 | EPI_ISL_1751669 | B.1.221 |
| **V2093548** | 2020-10-22 | EPI_ISL_1751658 | B.1.235 |
| **V20102415** | 2020-11-22 | EPI_ISL_1751164 | B.1.1.29 |
| **V20102263** | 2020-11-22 | EPI_ISL_1751162 | B.1.1.29 |
| **V2098433** | 2020-11-08 | EPI_ISL_1751771 | B.1.1 |
| **V2098054** | 2020-11-06 | EPI_ISL_1751766 | B.1.1 |
| **V20102989** | 2020-11-24 | EPI_ISL_1751182 | B.1.1.70 |
| **V20100597** | 2020-11-16 | EPI_ISL_1751101 | B.1.1.70 |
| **V2095479** | 2020-10-28 | EPI_ISL_1751693 | B.1.1.70 |
| **V2098442** | 2020-11-08 | EPI_ISL_1751772 | B.1.1.70 |
| **V2060716** | 2020-11-03 | EPI_ISL_1751513 | B.1.1.70 |
| **V2097556** | 2020-11-04 | EPI_ISL_1751754 | B.1.1.70 |
| **V2098994** | 2020-11-10 | EPI_ISL_1751778 | B.1.1.74 |
| **V2097325** | 2020-11-03 | EPI_ISL_1751752 | B.1.1.70 |
| **V20101616** | 2020-11-19 | EPI_ISL_1751133 | B.1.1.70 |
| **V2093455** | 2020-10-22 | EPI_ISL_1751657 | B.1.1.70 |
| **V20101505** | 2020-11-18 | EPI_ISL_2095200 | B.1.1.70 |
| **V20101818** | 2020-11-19 | EPI_ISL_1751145 | B.1.1.70 |
| **V2099507** | 2020-11-11 | EPI_ISL_1751788 | B.1.1.70 |
| **V2096671** | 2020-11-02 | EPI_ISL_1751737 | B.1.1.70 |
| **V2064501** | 2020-11-24 | EPI_ISL_1751553 | B.1.1.70 |
| **V20101648** | 2020-11-19 | EPI_ISL_1751136 | B.1.1.70 |
| **V20101320** | 2020-11-18 | EPI_ISL_1751124 | B.1.1.70 |
| **V20100480** | 2020-11-15 | EPI_ISL_1751096 | B.1.1.70 |
| **V20100354** | 2020-11-15 | EPI_ISL_1751086 | B.1.1.70 |
| **V20106092** | 2020-11-06 | EPI_ISL_1751278 | B.1.1.70 |
| **V20107272** | 2020-12-09 | EPI_ISL_1751327 | B.1.1.70 |
| **V20100482** | 2020-11-15 | EPI_ISL_1751098 | B.1.1.70 |
| **V20108782** | 2020-12-10 | EPI_ISL_1751343 | B.1.1.70 |
| **V20105762** | 2020-12-04 | EPI_ISL_1751265 | B.1.1.317 |
| **V20108521** | 2020-12-10 | EPI_ISL_1751336 | B.1.1.288 |
| **V2094680** | 2020-10-26 | EPI_ISL_1751670 | B.1.1.277 |
| **V20103576** | 2020-11-26 | EPI_ISL_1751209 | B.1.1.277 |
| **V20106044** | 2020-12-06 | EPI_ISL_1751277 | B.1.1.277 |
| **V20109225** | 2020-12-13 | EPI_ISL_1751359 | B.1.1.277 |
| **V2094154** | 2020-10-24 | EPI_ISL_1751665 | B.1.1.277 |
| **V2096155** | 2020-10-30 | EPI_ISL_1751717 | B.1.1.277 |
| **V2095673** | 2020-10-29 | EPI_ISL_1751699 | B.1.1.277 |
| **V2093947** | 2020-10-23 | EPI_ISL_1751662 | B.1.1.277 |
| **V2088895** | 2020-10-06 | EPI_ISL_1751630 | B.1.1 |
| **V2098035** | 2020-11-06 | EPI_ISL_1751765 | B.1.177 |
| **V2055095** | 2020-09-29 | EPI_ISL_1751502 | B.1.177 |
| **V2086733** | 2020-09-29 | EPI_ISL_1751617 | B.1.177 |
| **V20108856** | 2020-12-11 | EPI_ISL_1751346 | B.1 |
| **V20109308** | 2020-12-13 | EPI_ISL_1751361 | B.1.1.141 |
| **V20105258** | 2020-12-02 | EPI_ISL_1751257 | B.1.1.141 |
| **V20107167** | 2020-12-09 | EPI_ISL_1751323 | B.1.1.141 |
| **V20109762** | 2020-12-14 | EPI_ISL_1751375 | B.1.1.141 |
| **V20109799** | 2020-12-14 | EPI_ISL_1751379 | B.1.1.141 |
| **V2088911** | 2020-10-06 | EPI_ISL_1751631 | B.1.1.74 |
| **V20101615** | 2020-11-19 | EPI_ISL_1751132 | B.1.1.39 |
| **V20109072** | 2020-12-11 | EPI_ISL_1751353 | B.1.1.39 |
| **V20100104** | 2020-11-13 | EPI_ISL_1751075 | B.1.1.39 |
| **V2091084** | 2020-10-14 | EPI_ISL_1751638 | B.1.1.39 |
| **V2099369** | 2020-11-11 | EPI_ISL_1751785 | B.1.1.39 |
| **V20108790** | 2020-12-11 | EPI_ISL_1751345 | B.1.1.39 |
| **V2067371** | 2020-12-12 | EPI_ISL_1751579 | B.1.1.39 |
| **V20103218** | 2020-11-25 | EPI_ISL_1751198 | B.1.1.74 |
| **V2088152** | 2020-10-04 | EPI_ISL_1751622 | B.1.1.232 |
| **V20106996** | 2020-12-08 | EPI_ISL_1751319 | B.1.1.297 |
| **V20103465** | 2020-11-26 | EPI_ISL_1751203 | B.1.1.297 |
| **V20105782** | 2020-12-04 | EPI_ISL_1751266 | B.1.1.29 |
| **V2097895** | 2020-11-05 | EPI_ISL_1751763 | B.1.1.74 |
| **V2095973** | 2020-10-30 | EPI_ISL_1751710 | B.1.1 |
| **V2099661** | 2020-11-11 | EPI_ISL_1751797 | B.1.1.74 |
| **V20101890** | 2020-11-20 | EPI_ISL_1751148 | B.1.1.29 |
| **V20109309** | 2020-12-13 | EPI_ISL_1751362 | B.1.1.29 |
| **V20102745** | 2020-11-23 | EPI_ISL_1751170 | B.1.1.29 |
| **V2097689** | 2020-11-05 | EPI_ISL_1751758 | B.1.1.74 |
| **V20101995** | 2020-11-20 | EPI_ISL_1751153 | B.1.1.29 |
| **V2063654** | 2020-11-19 | EPI_ISL_1751538 | B.1.1.29 |
| **V2098683** | 2020-11-09 | EPI_ISL_1751774 | B.1.1.140 |
| **V20109744** | 2020-12-14 | EPI_ISL_1751372 | B.1.1.140 |
| **V20109007** | 2020-12-11 | EPI_ISL_1751350 | B.1.1.140 |
| **V20102935** | 2020-11-24 | EPI_ISL_1751180 | B.1.1.140 |
| **V2067861** | 2020-12-15 | EPI_ISL_1751583 | B.1.1.140 |
| **V20103499** | 2020-11-26 | EPI_ISL_1751206 | B.1.1.140 |
| **V20106871** | 2020-12-08 | EPI_ISL_1751311 | B.1.1.140 |
| **V20109433** | 2020-12-13 | EPI_ISL_1751364 | B.1.1.140 |
| **V20109053** | 2020-12-12 | EPI_ISL_1751352 | B.1.1.140 |
| **V20104012** | 2020-11-28 | EPI_ISL_1751219 | B.1.1.140 |
